# UNCOVERING DEEPER MEANING: APPLYING MULTIPLE METHODS OF DATA ANALYSIS IN NARRATIVE INQUIRY WITH OLDER ADULTS

**DOI:** 10.1093/geroni/igz038.2845

**Published:** 2019-11-08

**Authors:** Rebecca Meraz, Kathryn Osteen, Jocelyn S McGee

**Affiliations:** 1 Baylor University Louise Herrington School of Nursing, Dallas, Texas, United States; 2 Baylor University, Dallas, Texas, United States; 3 Baylor University, Waco, Texas, United States

## Abstract

Personal narrative is at the heart of how humans share information, represent identity, and convey ideas. Using narrative inquiry, researchers may gain some insight into an individual’s personal understanding of the events in their life. Although many narrative investigations report themes from study data, there is no single, well defined approach to data analysis in narrative research. We describe how applying multiple methods of systematic evaluation to narrative data leads to a deeper and more holistic understanding of told stories about medication-taking decisions among older heart failure (HF) patients. In this paper, we share a more holistic interpretation of the decision making process by applying Riessman’s thematic, structural, and performance analysis provided a method for analyzing the data beyond the spoken words. Data was transcribed verbatim then arranged according to the essential discourse between the participant and researcher, the intact story, and the story elements needed to facilitate each method of evaluation. The data analysis process outlined in this article contributes to the academic discourse and knowledge supporting the use of multiple methods of systematic evaluation to uncover deeper meaning in narrative data. In clinical practice, care should be guided by a deeper and holistic understanding of the decisions patients make about whether to take or not take a HF medication.

